# Targeting stem cells in myelodysplastic syndromes and acute myeloid leukemia

**DOI:** 10.1111/joim.13535

**Published:** 2022-07-13

**Authors:** Petter S. Woll, Tetsuichi Yoshizato, Eva Hellström‐Lindberg, Thoas Fioretos, Benjamin L. Ebert, Sten Eirik W. Jacobsen

**Affiliations:** ^1^ Department of Medicine Huddinge Center for Hematology and Regenerative Medicine Karolinska Institutet Stockholm Sweden; ^2^ Department of Hematology Karolinska University Hospital Stockholm Sweden; ^3^ Division of Clinical Genetics Department of Laboratory Medicine Lund University Lund Sweden; ^4^ Division of Laboratory Medicine Department of Clinical Genetics and Pathology Lund Sweden; ^5^ Department of Medical Oncology Dana–Farber Cancer Institute Boston Massachusetts USA; ^6^ Broad Institute of Harvard and MIT Cambridge Massachusetts USA; ^7^ Howard Hughes Medical Institute Boston Massachusetts USA; ^8^ Department of Cell and Molecular Biology Karolinska Institutet Stockholm Sweden; ^9^ MRC Molecular Haematology Unit MRC Weatherall Institute of Molecular Medicine University of Oxford Oxford UK

**Keywords:** acute myeloid leukemia, clonal evolution, hematopoietic stem cells, leukemic stem cells, myelodysplastic syndromes, therapeutic targets

## Abstract

The genetic architecture of cancer has been delineated through advances in high‐throughput next‐generation sequencing, where the sequential acquisition of recurrent driver mutations initially targeted towards normal cells ultimately leads to malignant transformation. Myelodysplastic syndromes (MDS) and acute myeloid leukemia (AML) are hematologic malignancies frequently initiated by mutations in the normal hematopoietic stem cell compartment leading to the establishment of leukemic stem cells. Although the genetic characterization of MDS and AML has led to identification of new therapeutic targets and development of new promising therapeutic strategies, disease progression, relapse, and treatment‐related mortality remain a major challenge in MDS and AML. The selective persistence of rare leukemic stem cells following therapy‐induced remission implies unique resistance mechanisms of leukemic stem cells towards conventional therapeutic strategies and that leukemic stem cells represent the cellular origin of relapse. Therefore, targeted surveillance of leukemic stem cells following therapy should, in the future, allow better prediction of relapse and disease progression, but is currently challenged by our restricted ability to distinguish leukemic stem cells from other leukemic cells and residual normal cells. To advance current and new clinical strategies for the treatment of MDS and AML, there is a need to improve our understanding and characterization of MDS and AML stem cells at the cellular, molecular, and genetic levels. Such work has already led to the identification of promising new candidate leukemic stem cell molecular targets that can now be exploited in preclinical and clinical therapeutic strategies, towards more efficient and specific elimination of leukemic stem cells.

## Graphical abstract

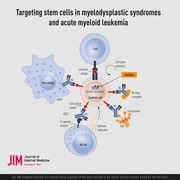

## Introduction

The recognition that cellular, genetic, and molecular features of cancer allow for segregation of cancer subgroups with different diagnosis, disease progression, and response to treatment, has facilitated development of therapeutic strategies tailored towards patients most likely to respond while reducing the risk of life‐threatening side effects [1, 2]. Advances in next‐generation sequencing have brought this down to the single cell level, and for many cancerous conditions we now have a detailed overview of the genetic architecture with identification of highly recurrent genetic lesions, as well as molecular profiles based on gene expression, which segregate with different cancer diagnosis, prognosis, and treatment responses [3]. This has facilitated a more targeted assignment of clinical strategies for individual patients and allowed the development of new promising therapeutic approaches. However, the continued development of precision‐based medicine towards tailored individual treatment of cancer remains important because many well‐known molecular markers or disease drivers lack a matching drug, and treatment‐related morbidity and mortality still represent major challenges for many cancer groups. Understanding the cellular and molecular biology of relapse is likely to be critical as selective persistence of distinct cancer cells has been observed following treatment, which frequently seems to be linked to the selective resistance of cancer stem cells [4]. As the majority of conventional therapies have been designed to efficiently target the majority or bulk of cancer cells, rather than the cancer stem cells themselves, few treatment strategies have been developed to also efficiently target the cancer stem cells, which is required and potentially sufficient to achieve a cure. Part of the challenge towards this goal is that we, for many cancers, still do not know the exact identity of the cells representing the true cancer stem cells; therefore, we also do not know the molecular identity required to identify cancer stem cell therapeutic targets. Progress towards this goal remains hampered by the ability of available human stem cell assays to reliably read out cancer stem cells and of preclinical assays to reliably predict the therapeutic impact on cancer stem cells within patients [5, 6].

In light of the identity of cancer stem cells (like their normal counterparts) being defined by their functional characteristics, significant efforts have been directed towards the development of experimental assays for assessment of self‐renewal and tumor‐propagation potentials from human patient material [5]. Combined with the easy accessibility of hematopoietic tissue and already defined distinct hematopoietic stem and progenitor cells within the bone marrow of normal healthy individuals, hematologic malignancies have been at the forefront of studies aiming to identify and characterize cancer stem cells and tumor propagating cells. While the first experimental evidence supporting the existence of cancer stem cells was obtained from patients with acute myeloid leukemia (AML) following transplantation into immune‐compromised mice [7, 8], it remains unclear to what degree these assays reliably detect all AML propagating cells and stem cells [9, 10]. While these assays nevertheless remain important in studies of cancer stem cells, this highlights the importance of also developing strategies facilitating the identification, fate mapping, and clinical surveillance of cancer stem cells within the patient at diagnosis, disease progression, and in response to treatment.

Myelodysplastic syndromes (MDS) and AML are two related myeloid malignancies, which each encompass a heterogeneous group of patients with regard to clinical features, genetics, prognosis, and treatment [11, 12]. The advanced age of most MDS and AML patients presents challenges and limitations to the treatment strategies, as these patients often do not tolerate the same treatments as applied to younger individuals. Genetic profiling has demonstrated that MDS and AML patients share many recurrent genetic driver lesions [13, 14, 15, 16], and in addition to de novo AML, many MDS patients progress to AML (secondary AML) [17]. Furthermore, the recent discovery of clonal hematopoiesis (CH), characterized by the detection of recurrent genetic lesions also associated with MDS and AML, in a significant proportion of the blood cells from healthy individuals, confers clonal expansion and an increased risk for later clonal evolution and development of MDS or AML, in line with a multihit process for leukemic transformation and progression. In this short review, we review our current knowledge with regard to the cellular origin of leukemic stem cells in MDS and AML, as well as their relevance to current and future therapeutic strategies.

## Identification and characterization of MDS and AML stem cells

**Fig. 1:**
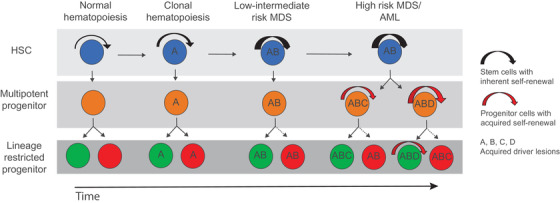
Clonal evolution within distinct hematopoietic stem and progenitor cell compartments. Normal hematopoietic stem cells (HSCs) do not have any detectable driver mutations. In clonal hematopoiesis (CH), a recurrent driver mutation is acquired. Findings suggest that to be sustained in the long term the CH mutation must be targeted to HSCs possessing extensive self‐renewal potential. In low‐to‐intermediate‐risk myelodysplastic syndromes (MDS), additional driver mutations are acquired, and these must also typically be targeted to normal or CH stem cells to be sustained over time, as these mutations will not introduce the self‐renewal ability to downstream progenitors. In contrast, in progressed high‐risk MDS and transformed acute myeloid leukemia (AML), long‐term self‐renewal potential also appears to have been acquired by downstream progenitors, and therefore new mutations might initially have been targeted towards a progenitor rather than an HSC.

Leukemic transformation is driven by the sequential acquisition of genetic driver lesions targeted towards hematopoietic stem and progenitor cell compartments. With a few notable exceptions, multiple genetic driver lesions are required for leukemic transformation [18]. The application of high‐throughput DNA sequencing has demonstrated that a large proportion of healthy elderly individuals with normal blood values carry recurrent somatically acquired mutations in blood cells associated with hematologic malignancies, including AML and MDS [19, 20]. These mutations, which frequently are predicted as initiating mutations in AML and MDS, are associated with increased risk for later transformation to myeloid malignancies, and this condition is in healthy individuals referred to as CH. The identity of the normal hematopoietic stem and progenitor cells to which these initiating and transforming mutations are targeted is likely to be important for the transformation risk. By performing whole genome profiling of clones derived from single cells in normal subjects with CH [21] and in patients with hematologic malignancies [22], and using the natural accumulation of genomic mutations as a measure to estimate the timing of acquisition of the driver mutations, it has been demonstrated that these CH mutations are often acquired decades before the CH clone becomes detectable [21, 22, 23, 24], and in some cases can even be tracked to embryonic development [22]. In order for a somatically acquired lesion to persist and the clone to expand for such a long period in a tissue associated with a high degree of cellular turnover such as the hematopoietic system, it is critical that the cell to which the mutation was targeted either already possesses extensive self‐renewal potential prior to acquisition or acquires self‐renewal potential as a result of the mutation. In the normal human hematopoietic hierarchy, hematopoietic stem cells (HSCs) endowed with life‐long self‐renewal potential give rise to downstream, more short‐lived progenitor cells (Fig. 1). Based on genomic profiling of single cell–derived colonies combined with computational analysis, it has been predicted that a healthy adult individual has 50,000–200,000 HSC clones that actively contribute to hematopoiesis through the hierarchical generation of downstream progenitor cells (Fig. 1) [25]. The combined application of flow cytometry with in vitro and in vivo HSC assays has suggested that human HSCs are confined to the lineage−CD34+CD38−CD90+CD45RA− compartment [26, 27]. Acquisition of a CH recurrent driver mutation confers clonal fitness and promotes clonal expansion of mutated cells [23], but in isolation this is not sufficient to cause transformation to myeloid malignancies. Rather, in healthy individuals, this gives rise to CH, resulting in clonal expansion but with little or no impact on normal blood lineage replenishment. Analysis of CH mutations in mature blood cell lineages has suggested that the CH mutations are targeted to a multipotent (lymphomyeloid) stem/progenitor cell [28], and other studies have shown in a limited number of cases that this typically reflects that the CH mutations can be detected in the lineage−CD34+CD38−CD90+CD45RA− HSC compartment [29], implying that CH driver mutations targeted towards downstream progenitor cells are either lost due to limited potential for self‐renewal or that CH clones originating from progenitors do not reach a clonal size that can be detected with the current available methods (Fig. 1). Although most of the cases with CH have normal blood values, individuals with CH do have a higher propensity for transformation to myeloid malignancies [19, 20]. Although the specific gene mutated and the size of the CH clone, as reflected in the variant allele frequency (VAF), are correlated to the risk for transformation, it remains difficult to predict which individuals with CH are likely to transform, and the overall risk for leukemic transformation remains low in individuals with CH [19, 30]. A direct link between the establishment of preleukemic stem cells and subsequent leukemic transformation can also be observed in de novo AML. *DNMT3A*, the most commonly mutated gene in CH individuals, is also recurrently mutated in AML patients where *DNMT3A* is frequently predicted to be the first/initiating event [31]. Through analysis of the clonal involvement in distinct hematopoietic stem and progenitor cell compartments, as well as mature blood cell lineages, preleukemic clones containing the *DNMT3A* mutation but not the other driver mutations detected in the AML cells have been tracked to the HSC compartment [31, 32], and unlike the transformed AML cells, these preleukemic cells contribute to both myeloid and lymphoid lineages [31], again suggesting preserved hematopoietic functions prior to acquisition of transforming events.

Upon acquisition of a transforming event in a CH clone, this can give rise to MDS or de novo AML. Low‐to‐intermediate‐risk MDS with a relatively low frequency of leukemic blasts preserves phenotypically, molecularly, and functionally distinct hematopoietic stem and progenitor cell compartments [33, 34, 35, 36]. By applying the same cell surface antigens used to identify distinct stem and progenitor cells in normal bone marrow, it is therefore possible to specifically isolate phenotypically, molecularly, and functionally distinct lineage‐restricted myeloid and erythroid progenitors and HSCs that are all highly clonally involved [34]. These findings, as well as hierarchical in vivo experiments with bone marrow from patients that engraft in immune‐deficient mice [34] imply a unidirectional cellular hierarchy in low‐to‐intermediate‐risk MDS, where lineage−CD34+CD38−CD90+CD45RA− MDS stem cells give rise to clonally involved lineage‐restricted myeloid and erythroid progenitors [34]. This, combined with the finding that identified recurrent genomic lesions in low‐to‐intermediate‐risk MDS patients are consistently tracked back to their lineage−CD34+CD38−CD90+CD45RA− MDS stem cell compartment, imply not only that these genomic lesions have uniformly been targeted to the rare HSC compartment, but also that only HSCs can propagate and in the long term sustain the MDS clone in low‐to‐intermediate‐risk MDS patients (Fig. 1). Furthermore, this also suggests that despite a high clonal involvement in downstream MDS progenitor cells, as revealed by a high VAF, the recurrent genetic lesions acquired in low‐to‐intermediate‐risk MDS are not sufficient to confer self‐renewal potential outside the HSC compartment, similar to individuals with CH.

In distinction to low‐to‐intermediate‐risk MDS, upon progression to high‐risk MDS and AML, recurrent driver mutations cannot always be accounted for within the lineage−CD34+CD38−CD90+CD45RA− HSC compartment, suggesting that self‐renewal and leukemic propagating potential has been extended to cells outside the phenotypic HSC compartment (Fig. 1) [31, 37, 38]. Furthermore, the ability to propagate AML in immune‐compromised mice has been observed in both the phenotypic stem and progenitor cell compartments [39, 40]. The interpretation of these findings in the case of advanced MDS and AML could however be confounded by aberrant phenotypic and molecular signatures of clonally involved stem and progenitor cells.

Several normal cell surface antigens, such as CD33 [41, 42], CD123 [43, 44], CD47 [45, 46], CD371 (CLL‐1 or CLEC12A) [47, 48], CD70 [49], CD366 (TIM3) [50, 51], and IL1RAP [52, 53, 54], have been found to be either more highly expressed or to display selective expression on MDS and AML leukemia propagating cells; but it remains unclear to what degree they can reliably identify the entire leukemia stem cell population. In addition to their potential to self‐renew, MDS and AML stem cells display a high degree of cellular quiescence. This dormant and metabolically inactive state has been implicated in the ability of MDS and AML stem cells to selectively evade therapeutic targeting [55, 56]. It is likely that MDS and AML stem cells also possess other unique properties to escape therapeutic targeting as well as immune surveillance [57, 58, 59]. Thus, revealing the identity of the cells responsible for leukemic initiation and propagation is critical to understanding the cellular and molecular basis for relapse and allowing the identification of new therapeutic targets and strategies, as efficient elimination of MDS and AML stem cells will be a requisite for the development of treatments with a higher curative potential.

## Clinical surveillance of stem cells in MDS and AML

**Fig. 2:**
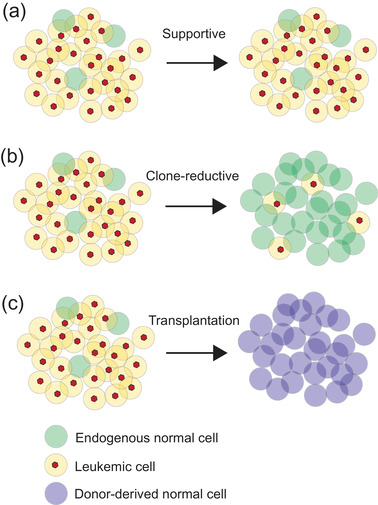
Current common therapeutic approaches for myelodysplastic syndromes (MDS) and acute myeloid leukemia (AML). The treatment of MDS and AML depends on patient and disease characteristics. Under supportive treatment (a), normal hematopoietic functions can be improved without significantly reducing the leukemic burden by infusion of mature blood cells (red blood cells, platelets) or stimulation of hematopoietic differentiation by cytokines such as EPO, G‐CSF, or TPO‐mimetics. Clone reductive treatment (b) allows for reduction of the leukemic burden, either by preferential elimination of clonally involved cells or targeting of both normal and leukemic cells. Following transplantation (c), endogenous normal and leukemic cells are replaced by normal donor‐derived hematopoietic cells.

Dependent on the patient characteristics and disease subtype, current MDS and AML treatment options can be divided into supportive treatment, clone‐reductive treatment, and treatment aiming for cure, including induction chemotherapy supported by targeted treatment for certain forms of AML and allogeneic hematopoietic stem cell transplantation (SCT) for other AML and all MDS (Fig. 2) [11]. The heterogeneous nature of both MDS and AML presents clinical challenges when selecting appropriate treatment options, including wait‐and‐see strategies for certain patients [60]. In particular, the old age and frailty of many patients reduce the availability of the treatment options that can safely be administered while at the same time promoting the intended clinical effect to either stabilize, reduce, or eliminate the disease burden.

As incorporated into the diagnostic criteria, cytopenia impacting one or more of the erythroid, myeloid, or platelet lineages is a common feature in MDS, with anemia being the most prevalent, present in 80% of patients [61, 62]. Pancytopenia may be present at diagnosis in almost half of the patients and usually deteriorates with the course of the disease [61, 62]. Cytopenia is also often observed in AML, especially in secondary cases preceded by MDS [63]. Although recurrent transfusions constitute basic supportive care for anemia, this leads with time to iron overload and organ damage unless iron chelation treatment is initiated [64, 65, 66]. Treatments with cytokines supporting the generation of mature blood cell lineages have shown effectiveness in several patient groups, including use of erythropoietin (EPO) [67, 68], granulocyte colony stimulating factor (G‐CSF) [69], and thrombopoietin (THPO and THPO‐mimetics) [70, 71, 72]. However, whether the clinical benefit of these supportive strategies represents a direct effect on clonally involved cells or originates from residual nonclonally involved cells remains unclear. Furthermore, as these treatment strategies fail to reduce the clonal involvement of the malignant clone or reduce the MDS‐ or AML‐propagating cells, they do not have curative potential but remain important strategies to alleviate common symptoms and prolong the time to onset of permanent transfusion need [67].

Although conventional chemotherapy has been used to treat high‐risk MDS [73, 74], its effect on survival is limited and hypomethylating agents are now the preferred first‐line treatment option in high‐risk MDS cases not eligible for allogeneic SCT and is even frequently used as a bridge to transplant [11, 75]. In MDS and AML, genes related to epigenetic regulation including *DNMT3A*, *TET2*, and *ASXL1* are frequently mutated along with mutations in splice factor genes [13, 14, 15, 16]. Inhibiting DNA methylation through the hypomethylating agents 5‐azacitidine or decitabine can prolong survival and improve the quality of life compared to conventional chemotherapy [76, 77, 78]. A BCL‐2 inhibitor, venetoclax, is approved in combination with 5‐azacitidine or decitabine to treat newly diagnosed AML patients both in the United States and Europe [79]. Through BCL‐2 inhibition, venetoclax induces apoptosis of tumor cells. Lenalidomide, a derivative of thalidomide, has a variety of effects including immunomodulation and is an option for isolated del(5q) MDS at EPO failure, provided patients do not carry *TP53* mutations [80, 81, 82].

Clone‐reductive therapies are critical strategies that can stabilize the disease, delay disease progression, and reduce the disease burden over a longer period, thereby prolonging the quality of life and lifespan. Such effects can be achieved by therapy that targets both bulk and leukemic stem cells, as well as by therapy that targets the bulk leukemia preferentially, where the latter would lead to a likely relapse. In addition to hypomethylating agents, treatments targeting genetic features of the malignant clone, such as FLT3 inhibitors (*FLT3* mutated AML) and IDH1/IDH2 inhibitors (*IDH1*/*IDH2* mutant AML) are under development or in clinical use [83]. These approaches can induce long‐term clinical remission but are also associated with frequent relapses. Cytoreductive strategies can also be applied as a preconditioning strategy to reduce the clonal burden prior to allogeneic SCT. In combination with aggressive chemotherapy to eliminate normal and malignant bone marrow cells in the recipient patient, the allogeneic donor graft that restores normal hematopoiesis is associated with graft‐versus‐leukemia effect where donor‐derived immune cells provide an immune surveillance effect against residual malignant cells [84]. The importance of the graft‐versus‐leukemia effect is highlighted by the higher relapse rate observed in transplants where the donor cells originate from the monozygotic twin of the recipient [85]. In addition to balancing the graft‐versus‐leukemia effect against the general alloreactivity against host cells in other tissues resulting in graft‐versus‐host disease, the aggressive chemotherapy regimen applied to reduce the clonal burden is often not suited for MDS and AML patients due to their advanced age. As a result, several reduced intensity conditioning regimens have been explored [86, 87], and SCT has been made available as a treatment option for a higher number of MDS and AML patients. The graft‐versus‐leukemia effect is more critical in patients who received SCT with reduced‐intensity conditioning. However, also following SCT, the relapse rate remains high for both MDS and AML [88, 89]. and in combination with the risks associated with the SCT including graft‐versus‐host disease, infection, and organ failures [84], this highlights the importance of raising our understanding of the underlying disease biology and developing new and better treatment.

**Fig. 3:**
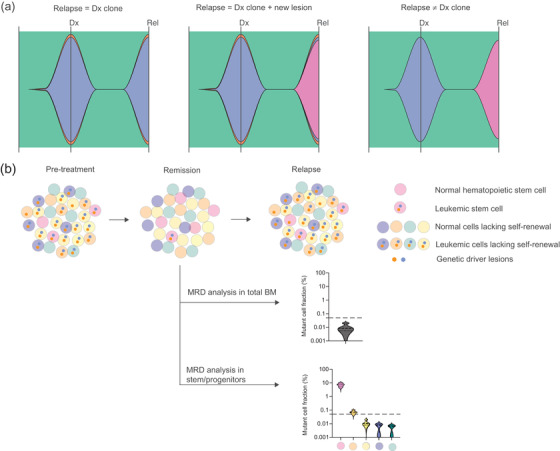
Genetic and cellular prediction of relapse. (a) Genetic composition of the malignant clone at diagnosis (Dx) and relapse where relapse is composed of the same dominant clone found at diagnosis (left), relapse originates from the clone dominating at diagnosis but with acquisition of a new genetic lesion (middle), and relapse originating from a clone not detected at diagnosis (right). (b) Treatment resulting in remission and subsequent relapse where relapse originates from a selectively persistent leukemic stem cell population at remission. The graphs illustrate the enhanced sensitivity of minimal residual disease (MRD) detection through targeted stem/progenitor cell analysis at remission.

By comparative genetic analysis of the dominating malignant clone before treatment and at relapse, three main patterns of relapse have emerged [55, 90–92]. In several cases, the relapse clone is genetically identical to the dominating clone detected prior to treatment or originates from the dominating clone prior to treatment but has acquired additional driver mutations (Fig. 3a). In rare cases, relapse in MDS and AML can also originate from a minor clone not detected by conventional sequencing methods prior to treatment. This suggests that for most MDS and AML patients, sensitive tracking of mutations detected prior to treatment, when the patient is in treatment‐induced remission, represents useful tools for monitoring minimal residual disease and potential early detection of relapse. However, in several studies where the malignant clone has been monitored by targeted DNA sequencing of bone marrow or peripheral blood post treatment, these strategies have so far been associated with a high degree of false negative findings, likely reflecting the infrequent nature of residual MDS or AML cells with relapse‐initiating properties in remission bone marrow or blood [93]. Furthermore, early detection of persistent mutant cells is also observed in patients who, under the length of the study, did not relapse, raising questions about the reliability of the predictive nature when residual malignant cells are detected shortly after treatment initiation [93]. The incorporation of mutational tracking following treatment to clinical routine therefore still awaits validation from large‐scale clinical studies.

The frequent relapses following treatment‐induced remission, which are often not predictable by sensitive mutational analysis of whole bone marrow or blood cells, implicates the persistence of rare, relapse‐initiating MDS or AML stem cells, with capacity to propagate the malignant clone (Fig. 3b) [94]. Indeed, following treatment‐induced remission by lenalidomide, identified as particularly effective in MDS patients with an isolated deletion of the long arm of chromosome 5 (del[5q]) [95, 96], selective persistence of clonally involved linage−CD34+CD38−CD90+ MDS stem cells can be reliably detected [55]. In contrast to analysis of whole bone marrow and even specific analysis of the immediate downstream lineage‐restricted CD34+CD38+ progenitor compartment, high clonal involvement was detected in the del(5q) MDS stem cell compartment specifically (Fig. 3b), and in all cases, this preceded relapse, which occurred many months later [55]. This implies that the specific analysis of the MDS (or AML) propagating compartments following treatment could allow more sensitive and earlier detection of rare, relapse‐initiating cells that persist following treatment‐induced remission (Fig. 3b). Although not straightforward to apply to routine clinical practice, targeted analysis of the rare MDS and AML stem/progenitor cells could improve our future ability to evaluate therapeutic impact, predict relapse at an early stage, and provide intervention at an early timepoint, to prevent relapse and disease progression. Combined application of targeted analysis of distinct disease‐propagating cells with highly sensitive DNA technologies can have clinical use in the future. However, care must be taken with negative findings as sensitivity will be limited by the number of cells analyzed, as well as the detection limit of the method, where total absence of relapse‐initiating cells will be difficult to establish due to both technical limitations with assay sensitivity and the number of cells analyzed. Furthermore, whether or not minimal residual disease detection in distinct disease‐propagating compartments accurately predicts an impending relapse requires evaluation in larger clinical trials. In light of findings from analysis of whole bone marrow, care should be taken towards minimal residual disease detection at very early timepoints, as these could indicate either a declining malignant burden or persistent malignant cells, warranting evaluation in a second later sample.

As indicated above, a major challenge with the development of treatment strategies aimed at targeting the disease‐propagating and/or relapse‐initiating cells in MDS and AML is the limited understanding of the cellular and molecular mechanisms that distinguish these cells from the short‐lived mature progeny, as well as their normal counterpart. However, cellular quiescence and stemness has both been implicated in treatment evasion and disease prognosis [55, 56, 97, 98], and a number of cell surface markers have been found to be upregulated on MDS and AML cells [43, 45, 51, 52]. As outlined below, these represent candidate targets for novel therapeutic strategies.

## Targeting cell surface molecules in MDS and AML

To date, many cell surface markers have been shown to be aberrantly expressed on AML stem cells, whereas fewer have been described on MDS stem cells [10, 99]. A major reason for the lack of identification of specific markers on MDS stem cells, in particular in low‐to‐medium risk cases, likely is their similarity to normal HSC and the difficulties in reliable studying of such cells in immunodeficient mice, which has become the standard model to study AML stem cells and to evaluate treatment effects. The most widely studied cell surface markers, expressed on AML stem cells, include CD33 [41, 42], CD123 [43, 44], CD47 [45, 46], CD371 (CLL‐1 or CLEC12A) [47, 48], CD70 [49], CD366 (TIM3) [50, 51], and IL1RAP [52, 53, 54].

**Fig. 4:**
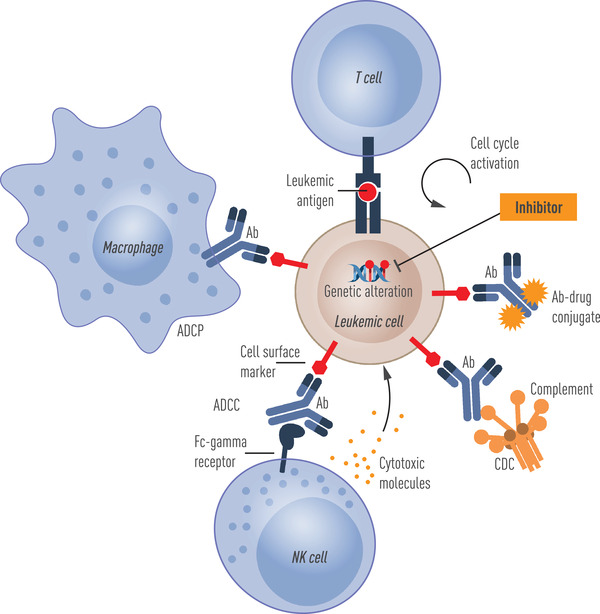
Exploiting cell surface and somatic genetic lesions for targeting of myelodysplastic syndrome– and/or acute myeloid leukemia–propagating cells. Genetic alterations in the form of DNA mutations or chromosome changes can allow the development of cellular and molecular therapeutics aimed specifically towards clonally involved cells. Leukemic cells can also acquire altered expression of normal cell surface molecules, which can be exploited therapeutically to activate complement‐dependent cytotoxicity (CDC), antibody‐dependent cellular cytotoxicity (ADCC), or antibody‐dependent cellular phagocytosis (ADCP), or be targeted by antigen‐specific T cells.

Cell surface markers provide ideal targets for treatment using various forms of recombinant antibodies (Fig. 4). Therapeutic antibodies can act through several different mechanisms; for example, they can be conjugated to a cytotoxic payload that upon internalization causes cell death, be engineered to either activate the innate immune system through complement‐dependent cytotoxicity, antibody‐dependent cellular phagocytosis by macrophages, or antibody‐dependent cellular cytotoxicity (ADCC) elicited by natural killer (NK) cells [100]. In addition, antibodies can be designed to simultaneously bind two antigens, so called bispecific antibodies, where bispecific T‐cell engagers bring the malignant stem cells in close proximity to T cells, thereby activating the adaptive immune system [101]. Additional, antibody constructs with multiple binding sites include dual‐affinity retargeting proteins, as well as bi‐ and tri‐specific killer engagers [101, 102].

To target a cell surface marker on an AML or MDS stem cell, these markers should ideally be expressed only on the malignant stem cell population and not on normal HSCs to allow replenishment of normal hematopoiesis following treatment. Furthermore, to reduce side effects, it is also important that the cell surface molecule is not expressed on cells in other vital tissues. The markers CD33 [41, 42], CD123 [43, 44], and CD47 [45, 46] are all expressed on normal HSC, albeit lower than on AML stem cells, with CD123 also displaying expression in endothelial cells [103]. CD371 (CLL‐1) [47, 48], CD366 (TIM3) [50, 51], and IL1RAP [104, 105] lack detectable expression on HSCs, making them particularly interesting as targets for antibody‐based treatment. Another aspect important to consider is whether a target on the cell surface marks the entire leukemia stem cell population or whether it becomes lost during treatment, thereby allowing regrowth of the leukemic stem cell population.

An often neglected factor when considering a cell surface marker as a therapeutic target is also whether the antigen is functionally important for the leukemic stem cell, for example, if it provides a receptor for a ligand that is important for its self‐renewal, survival, or capacity to evade the immune system. For example, CD47 is a cell surface antigen that is highly expressed on leukemic stem cells in many AML patients and mediates a don't‐eat‐me signal to cells of the innate immune system by interacting with the cognate SIRP1‐a receptor expressed on macrophages [45, 46]. This allows evasion from potential immune surveillance by the innate immune system. Blockade of the CD47‐SIRP1‐a interaction by an anti‐CD47 antibody has been shown to result in macrophage‐mediated phagocytosis of AML and MDS stem cells (Fig. 4) [45]. IL1RAP, the coreceptor of the IL1‐receptor (IL1R1), is necessary for conveying proinflammatory signals by IL‐1a and IL1b [106], of importance for the survival of primitive AML cells [54, 107]. IL1RAP is aberrantly expressed on AML stem cells in a great majority of patients and on high‐risk MDS stem cells [52, 105]. IL1RAP antibodies engineered to block IL1‐signaling and to enhance ADCC activity have shown strong therapeutic effects in patient‐derived chronic myeloid leukemia (CML) and AML xenograft models [53, 54].

The currently available preclinical models cannot be used to fully predict whether an antibody‐based therapy against a specific target on AML or MDS stem cells will be effective in a clinical setting, a major factor being the limitations of available in vitro and in vivo experimental models for MDS‐ and AML‐propagating cells. This necessitates early and often costly clinical development to assess their safety and efficacy in clinical trials. In addition, depending on the mode of action (MoA) of an antibody, combination therapies may be synergistic or detrimental; for example hypomethylating drugs have been shown to enhance the ADCC activity of NK cells, of importance if an antibody uses effector cells to elicit killing of their target cells [108], whereas intensive chemotherapy destroys immune cells that may be of importance depending on the MoA of the antibody. The latter also emphasizes the importance of considering the timing when an antibody is given in a treatment cycle with other drugs.

Of the cell surface markers discussed in previous paragraphs, only an antibody against CD33 (gemtuzumab) has received FDA approval [109]. A CD123‐directed cytotoxin (tagraxofusp), consisting of the fusion of interleukin‐3 with a truncated diphtheria toxin payload, recently received FDA approval for the treatment of blastic plasmacytoid dendritic cell neoplasm [110, 111] and clinical trials are ongoing in MDS and AML. A CD47 antibody (magrolimab) has received a Breakthrough Therapy designation by the United States Food and Drug Administration (FDA) for the treatment of newly diagnosed MDS [112]. Currently, a plethora of clinical trials at various phases are ongoing to assess the safety and clinical efficacy of antibodies directed at cell surface markers expressed on MDS and AML stem cells, with an increasing trend of using bispecific, immune cell engaging properties (www.clinicaltrials.gov). Many of these trials will read out in the near future, hopefully, leading to better treatment outcomes for patients diagnosed with MDS and AML and to increased insights into the biology of leukemia stem cells and the feasibility of using antibody based therapies directed at targets expressed on their cell surface.

Given the success of chimeric antigen receptor (CAR) T‐cell therapy mainly in B‐cell malignancies, there is an increasing interest to use this technology to also target MDS and AML stem cells. The fact that B‐cell malignancies are amenable to this approach is not surprising as several cell surface markers (e.g., CD19, CD22, BCMA) are highly specific for the B‐cell lineage and, with proper precautions and supportive treatment, an individual can live without normal B cells. In contrast, this is not the case for myeloid lineage as such cells are required to sustain the life of an individual. Many innovative approaches using the CAR‐T‐cell technology are currently being tested preclinically and in clinical trials [113]. Given the potency of CAR‐T‐cell technology, the selection of the antigen on the surface at which the CAR‐T‐cells are directed is even more critical than for antibody‐based treatment. Current clinical trials have used similar antigens on the surface of AML stem cells as used for antibodies, including CD33, CD123, and CLL‐1 (Fig. 4), but given the increased toxicity with this technology, there is a strong focus on developing CAR‐T‐cell therapy allowing for rapid on and off switching [113].

## Exploiting acquired genetic lesions as therapeutic targets in MDS and AML

Some of the recurrent somatic oncogenic mutations that drive MDS and AML provide unique opportunities for targeting the malignant cells with high specificity (Fig. 4). One class of therapeutics targets activated tyrosine kinases in myeloid malignancies [13, 114]. In particular, the development of inhibitors targeting the constitutive tyrosine kinase activity of FLT3 as a result of mutations targeted to the *FLT3* tyrosine kinase domain has been productive for the treatment of *FLT3*‐mutated AML [115]. In light of the growth and survival advantage mediated by constitutive active FLT3 signaling pathway, *FLT3*‐mutated AML cells are particularly sensitive towards targeted disruption [116, 117]. The emergence of new, improved FLT3‐inhibitors with less associated off‐target effects with potential to promote therapeutic effect even as a single agent are particularly promising. However, it will also be important for long‐term follow‐up studies to investigate the specific impact on the AML‐propagating cells. In the case of tyrosine kinase inhibitors utilized to target the *BCR::ABL1* fusion product in CML [117], mature leukemic cells are effectively eliminated, but quiescent CML stem cells selectively survive [118, 119, 120]. As a result, CML patients require long‐term treatment as discontinuation of the kinase inhibitor results in relapse originating from CML stem cells in the great majority of the patients [121].

Somatic mutations in *IDH1* and *IDH2* lead to reduced production of α‐ketoglutarate (α‐KG), the key product catalyzed by the normal IDH1/IDH2 enzymes, and production of a novel oncometabolite, 2‐hydroxygluterate (2‐HG) [122, 123]. Selective inhibitors for mutant IDH1 and IDH2 have been developed that restore enzymatic function and block production of the oncometabolite, 2‐HG [124, 125]. The IDH1 inhibitor, ivosidenib, and the IDH2 inhibitor, enasidenib, have been approved for the treatment of AML with mutations in the respective genes [126].

Heterozygous deletion of an essential gene can provide a therapeutic window to kill malignant cells, as is the case in del(5q) MDS patients treated with lenalidomide [95, 96]. Deletion of the long arm of chromosome 5 results in haplo‐insufficiency for many genes located within the common deleted region including *casein kinase 1A1* (*CSNK1A1*) [127]. Mouse studies targeting *Csnk1a1* demonstrated that although haplo‐insufficiency for CSNK1A1 promotes HSC expansion, in line with the competitive advantage of del(5q) MDS stem cells, homozygous deletion of *Csnk1a1* results in HSC failure [128]. Interestingly, lenalidomide, found particularly effective for promoting cytogenetic and clinical long‐term remission in del(5q) MDS, targets CSNK1A1 for E3 ubiquitin ligase‐mediated ubiquitination and degradation [129]. These findings explain the preferential impact of lenalidomide on del(5q) MDS cells already lacking one copy of *CSNK1A1*. However, lenalidomide is not sufficient to eliminate all del(5q) MDS stem cells, as a significant proportion remain even in the bone marrow of patients in complete clinical and cytogenetic remission [34].

Somatically acquired genetic lesions can alter cell state in a manner that makes leukemia cells susceptible to a pharmacologic intervention relative to normal cells, a concept termed synthetic lethality. This may be the basis of the activity of hypomethylating agents and venetoclax in myeloid malignancies [130, 131]. Mutations in components of the splicing machinery are observed in over 50% of MDS, resulting in aberrant splicing [132]. Ongoing studies aim to identify therapeutic agents targeting the spliceosome that could preferentially target the mutated cells [133, 134].

Somatically acquired mutations can also open future possibilities for immunological targeting of AML and MDS cells [135, 136, 137]. Mutations that alter the coding region of the gene, as well as mutations in core components of the splicing machinery resulting in aberrant transcripts, can result in the generation of neoantigens that are unique for the mutated cells, providing a potential opportunity for T cell or vaccine therapies.

A major challenge is that MDS and AML are genetically heterogeneous, and patients have multiple sequentially acquired mutations. Targeting a mutation acquired early in disease development would potentially eliminate all mutant cells, while targeting a mutation acquired late in disease ontogeny might only have an effect on a subclone. As revealed by targeting of the *BCR::ABL1* fusion with tyrosine kinase inhibitors in CML and del(5q) mutated cells with lenalidomide, the leukemic stem cells are particularly challenging to eliminate fully. Effective elimination of the leukemic stem cells will likely require combination therapies that carefully consider the duration, dose, and timing of treatment.

## Conclusions

Genomic profiling of MDS and AML has become clinical routine in many countries and to a large degree influences the clinical management of MDS and AML patients. This has led to the development of new promising therapies exploiting the altered genetic and cellular composition of MDS and AML, yet most patients today lack a treatment that is specifically designed for the underlying molecular cause of the disease. In addition, disease progression, relapse, and treatment‐related mortality still remain a challenge for the treatment of MDS and AML patients. Leukemic stem cells, required for the propagation of the leukemia, are often particularly challenging to eliminate and have evolved mechanisms to escape therapeutic targeting. New therapeutic strategies are, however, being tested in preclinical and clinical studies, including small molecules and antibody‐based therapies, hopefully improving our ability to specifically target the leukemic stem cells. The impact of treatment can be observed by tracking the leukemic stem cells in the patients following treatment, but in several cases this is limited by our ability to distinguish leukemic stem cells from other normal or leukemic cells and lack of sensitivity of available methods. Advances in single cell technology, combining genetic and molecular profiling of single cells can in the future facilitate better understanding of cells evading therapy, as well as allow clinical surveillance and detection of rare, therapy‐resistant leukemic stem cells prior to relapse.

## Conflict of interest

B. L. E. has received research funding from Celgene, Deerfield, Novartis, and Calico and consulting fees from GRAIL. B. L. E. is a member of the scientific advisory board and shareholder for Neomorph Therapeutics, TenSixteen Bio, Skyhawk Therapeutics, and Exo Therapeutics. T. F. is a cofounder and board member and owns shares in Cantargia AB. All the other authors have no conflict of interest to declare.

## Author contributions

Conceptualization: all authors; Writing – original draft: Petter S. Woll and Tetsuichi Yoshizato; Writing – review and editing: all authors.
